# Metabolic engineering of the l-serine biosynthetic pathway improves glutathione production in *Saccharomyces cerevisiae*

**DOI:** 10.1186/s12934-022-01880-8

**Published:** 2022-08-06

**Authors:** Jyumpei Kobayashi, Daisuke Sasaki, Kiyotaka Y. Hara, Tomohisa Hasunuma, Akihiko Kondo

**Affiliations:** 1 Graduate School of Science, Technology and Innovation, Kobe University, 1-1 Rokkodaicho, Nada-ku, Kobe, Hyogo 657-8501 Japan; 2 Graduate School of Nutritional and Environmental Sciences, University of Shizuoka, 52‑1 Yada, Suruga‑ku, Shizuoka, 422‑8526 Japan; 3 Engineering Biology Research Center, Kobe University, 1-1 Rokkodaicho, Nada-ku, Kobe, Hyogo 657-8501 Japan; 4 RIKEN Center for Sustainable Resource Science, 1-7-22 Suehiro-cho, Tsurumi-ku, Yokohama, Kanagawa 230-0045 Japan

**Keywords:** Glutathione, Yּeast, *Saccharomyces cerevisiae*, l-Serine, l-Cysteine, Glycine

## Abstract

**Background:**

Glutathione is a valuable tri-peptide that is industrially produced by fermentation using the yeast *Saccharomyces cerevisiae*, and is widely used in the pharmaceutical, food, and cosmetic industries. It has been reported that addition of l-serine (l-Ser) is effective at increasing the intracellular glutathione content because l-Ser is the common precursor of l-cysteine (l-Cys) and glycine (Gly) which are substrates for glutathione biosynthesis. Therefore, we tried to enhance the l-Ser biosynthetic pathway in *S*. *cerevisiae* for improved glutathione production.

**Results:**

The volumetric glutathione production of recombinant strains individually overexpressing *SER2*, *SER1*, *SER3*, and *SER33* involved in l-Ser biosynthesis at 48 h cultivation was increased 1.3, 1.4, 1.9, and 1.9-fold, respectively, compared with that of the host GCI strain, which overexpresses genes involved in glutathione biosynthesis. We further examined simultaneous overexpression of *SHM2* and/or *CYS4* genes involved in Gly and l-Cys biosynthesis, respectively, using recombinant GCI strain overexpressing *SER3* and *SER33* as hosts. As a result, GCI overexpressing *SER3*, *SHM2*, and *CYS4* showed the highest volumetric glutathione production (64.0 ± 4.9 mg/L) at 48 h cultivation, and this value is about 2.5-fold higher than that of the control strain.

**Conclusions:**

This study first revealed that engineering of l-Ser and Gly biosynthetic pathway are useful strategies for fermentative glutathione production by *S. cerevisiase*.

**Supplementary Information:**

The online version contains supplementary material available at 10.1186/s12934-022-01880-8.

## Background

Glutathione is the most abundant non-protein thiol compound in all living organisms [1]. Because of its important physiological functions including its ability to act as an antioxidant, a detoxifier of xenobiotics, and an immune booster [2–8], glutathione has been widely used in the medical, food and cosmetic industries [9, 10]. Therefore, the demand for glutathione has increased in recent years.

**Fig. 1:**
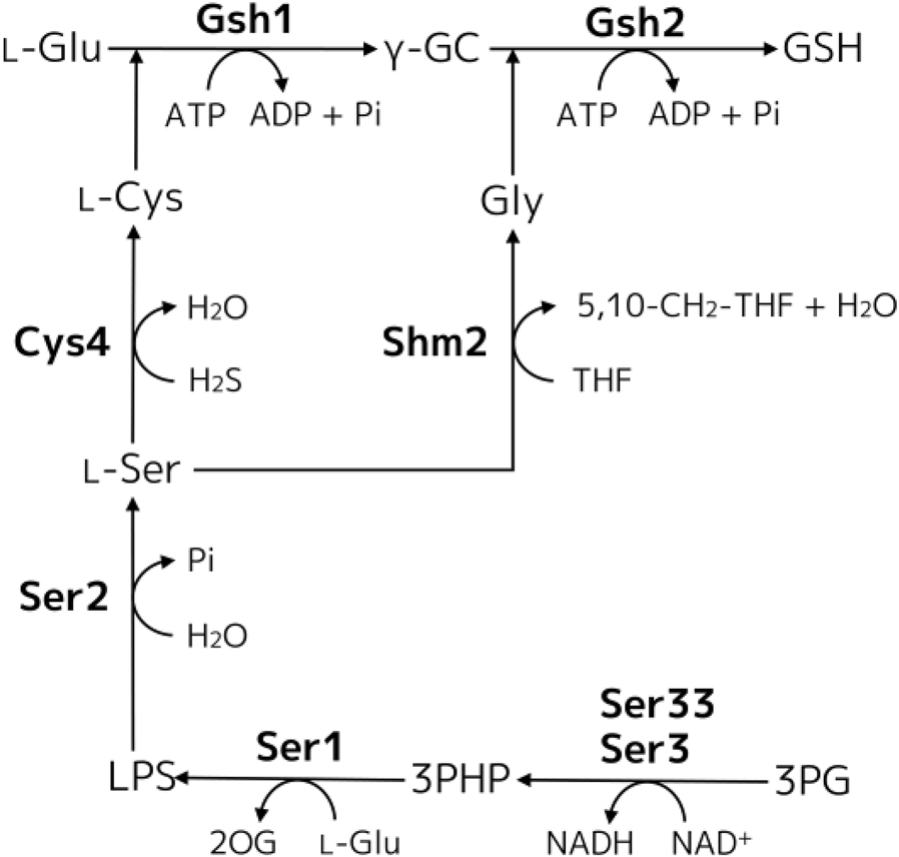
The metabolic pathway of the glutathione biosynthesis via l-Ser, l-Cys and Gly biosynthesis in *S. cerevisiae*. First, 3-phospho-glycerate (3PG) dehydrogenase encoded by *SER3* and *SER33* converts 3PG to 3-phospho-hydroxypyruvate (3PHP) using the oxidized form of nicotinamide adenine dinucleotide (NAD^+^) as a cofactor. Second, 3-phosphoserine aminotransferase encoded by *SER1* transfers an amino group from l-Glu to the 2-oxo group of 3PHP and produces l-*O*-phosphoserine (LPS) and 2-oxoglutarate (2OG). Finally, phosphoserine phosphatase encoded by *SER2* dephosphorylates LPS to l-Ser. The produced l-Ser is further metabolized to l-Cys and Gly by cystathionine β-synthase encoded by *CYS4* and serine hydroxymethyltransferase encoded by *SHM2*, respectively. Other abbreviations are as follows, 5,10-CH_2_-THF: (6R)-5,10-methylenetetrahydrofolate; *ADP*: adenosine diphosphate; *ATP*: adenosine triphosphate; *NADH*: reduced form of nicotinamide adenine dinucleotide; *Pi*: phosphate; *H*_*2*_*S*: hydrogen sulfide; *THF*: tetrahydrofolate.

At present, glutathione is produced mainly by fermentation using yeast. Glutathione biosynthesis is carried out by two consecutive adenosine triphosphate (ATP)-consuming reactions catalyzed by γ-glutamylcysteine (γ-GC) synthetase (GCS, EC 6.3.2.2), encoded by *GSH1*, and glutathione synthetase (GS, EC 6.3.2.3), encoded by *GSH2*, from three precursor amino acids, l-glutamate (l-Glu), l-cysteine (l-Cys), and glycine (Gly) (Fig. 1). GCS catalyzes the reaction to form γ-GC from l-Glu and l-Cys. GS catalyzes the reaction to form glutathione from γ-GC and Gly. Previously, we obtained a strain overexpressing multicopy of *GSH1* and *GSH2* genes (GCI strain) that showed increased glutathione production [11], and have used this GCI strain as a host [12–15].

Some studies have reported that supplying several types of amino acids during glutathione fermentation increased glutathione production in *Saccharomyces cerevisiae* [16, 17]. The addition of l-Cys was especially effective at increasing glutathione production, and metabolic engineering of l-Cys biosynthesis was also effective in enhancing glutathione production [11]. It was also reported that addition of l-serine (l-Ser) was effective in increasing the intracellular glutathione content [17] because l-Ser is the common precursor of l-Cys and Gly (Fig. 1). This fact suggests that intracellular l-Ser supplementation by genetic engineering should improve glutathione production without external addition of l-Ser. As shown in Fig. 1, l-Ser biosynthesis is carried out by three consecutive reactions from 3-phospho-glycerate (3PG), which is an intermediate of glycolytic pathway [18].

In this study, we enhanced glutathione productivity by metabolic engineering the l-Ser biosynthetic pathway from 3PG using the GCI strain as a host. Furthermore, we attempted to combine engineering of the l-Cys and Gly biosynthetic pathways with the l-Ser biosynthetic pathway to further enhance glutathione production.

## Results

### Effect of external l-Ser supplementation on glutathione production by GCI strain

**Fig. 2:**
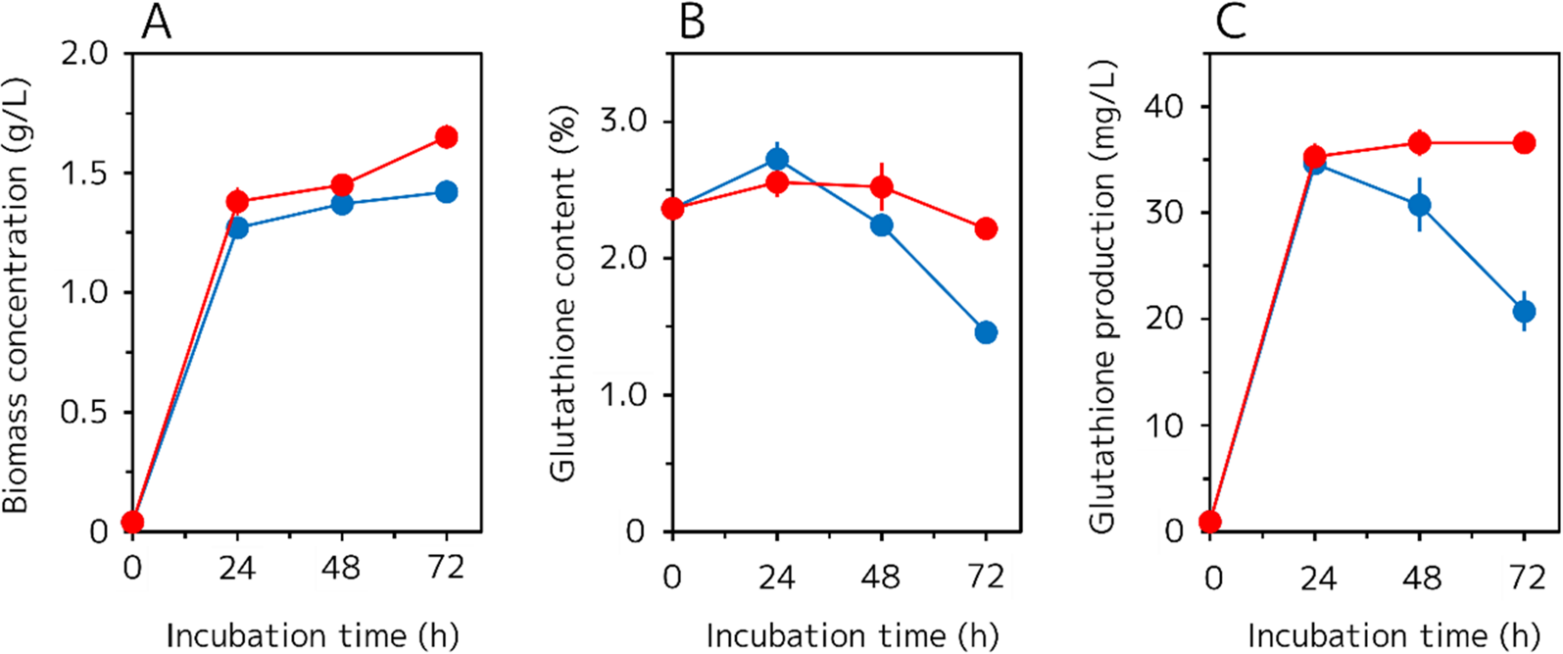
Time-dependent glutathione production of the GCI strain with or without external l-Ser supplementation. **A** Biomass concentration (g/L). **B** Intracellular glutathione content (%). **C** Volumetric glutathione production (mg/L). *Blue* and *red filled circles* represent the GCI strain grown without l-Ser and with 300 mg/L l-Ser, respectively. Values are the mean, and error bars show the standard deviation (n = 3)

l-Ser is a common precursor of both l-Cys and Gly, which are both substrates for glutathione biosynthesis (Fig. 1). To confirm the effect of external l-Ser supplementation on glutathione production in the GCI host strain, we measured biomass concentration (g/L), intracellular glutathione content (%), and volumetric glutathione production (mg/L) every 24 h for 72 h of fermentation with and without 300 mg/L l-Ser addition (Fig. 2). As shown in Fig. 2A, the biomass concentration was increased by addition of l-Ser (1.65 ± 0.05 g/L at 72 h) compared with no l-Ser addition (1.42 ± 0.03 g/L at 72 h). Furthermore, as shown in Fig. 2b, addition of l-Ser also increased the intracellular glutathione content (2.2 ± 0.1% at 72 h) compared with no l-Ser addition (1.5 ± 0.1% at 72 h) by about 1.5-fold. Because of the synergetic effect of the increased biomass concentration and intracellular glutathione content, the volumetric glutathione production was consistent for more than 72 h with l-Ser addition, whereas it was decreased when no l-Ser was added (Fig. 2C). However, the maximum yield of glutathione production was not enhanced by l-Ser addition at 24 h.

### Glutathione production by recombinant GCI strains engineered in l-Ser biosynthetic pathway

**Fig. 3:**
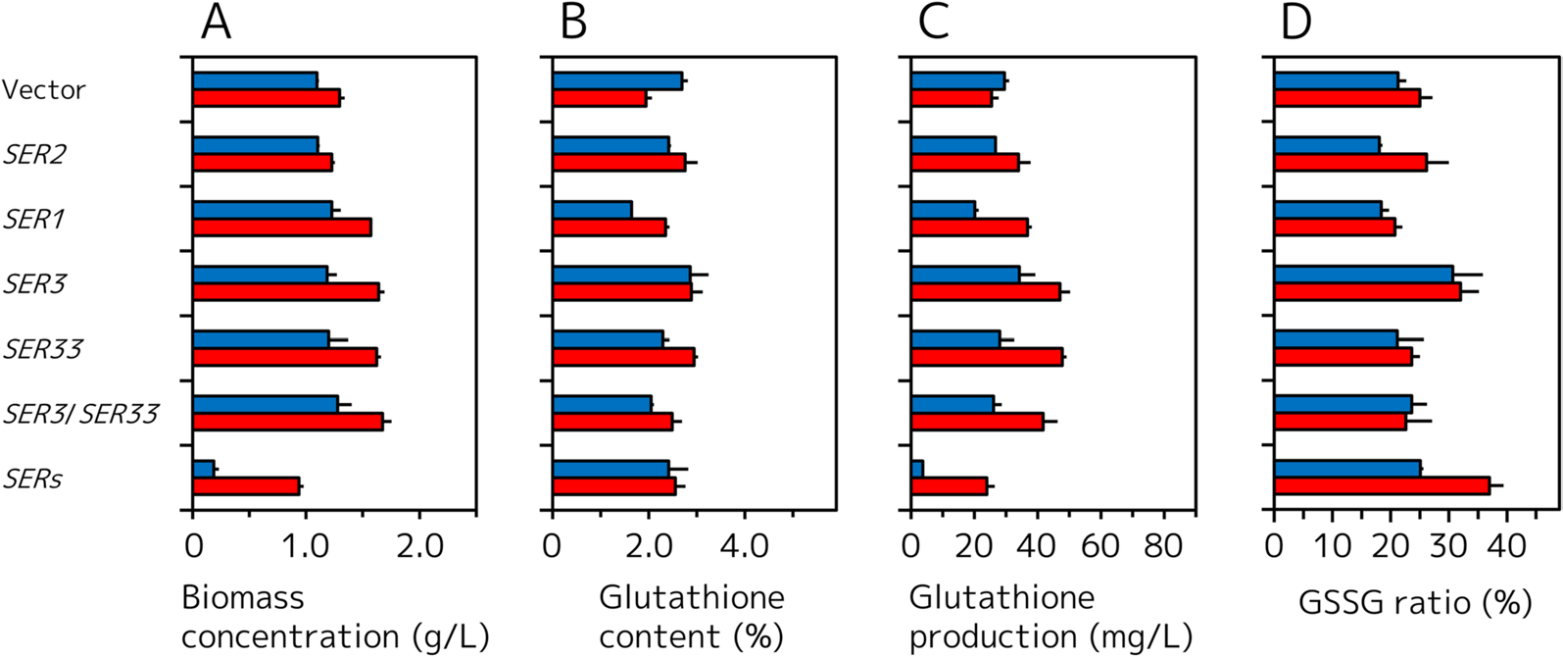
Glutathione production by GCI strains overexpressing l-Ser biosynthetic genes. **A** Biomass concentration (g/L). **B** Intracellular glutathione content (%). **C** Volumetric glutathione production (mg/L). **D** GSSG ratio (%). *Blue* and *red filled bars* represent the values after growth for 24 and 48 h, respectively. The GSSG ratio was defined as the ratio of the g-GSSG in cells to the g-total glutathione (GSH and GSSG) in cells. Values are the mean, and error bars show the standard deviation (n = 3)

L-Ser biosynthesis occurred through the l-Ser biosynthetic pathway from its precursor of 3PG, consistent with continuous reactions catalyzed by Ser2, Ser1, Ser3, and Ser33 (Fig. 1). To increase glutathione biosynthesis through enhancement of the l-Ser biosynthetic pathway, recombinant GCI strains overexpressing each gene (*SER2, SER1, SER3*, and *SER33*) and simultaneously overexpressing strains (*SER3*/*SER33* and *SER2*/*SER1*/*SER3*/*SER33*) were constructed, as well as a vector control GCI strain (Table 2). Figure 3 shows the biomass concentration, intracellular glutathione content, volumetric glutathione production, and GSSG ratio of these strains at 24 and 48 h. The biomass concentration of GCI/*SER2* (1.22 ± 0.03 g/L), GCI/*SER1* (1.57 ± 0.01 g/L), GCI/*SER3* (1.64 ± 0.05 g/L), GCI/*SER33* (1.62 ± 0.04 g/L), and GCI/*SER3*/*SER33* (1.67 ± 0.08 g/L) at 48 h were higher than that of the control GCI/Vector strain (1.12 ± 0.02 g/L), respectively (Fig. 3A). On the other hand, the biomass concentration of GCI/*SERs* (0.94 ± 0.04 g/L), which overexpresses *SER2*, *SER1*, *SER3*, and *SER33* at 48 h was decreased compared to that of the GCI/Vector strain. The intracellular glutathione content of the GCI/*SER2* (2.8 ± 0.8%), GCI/*SER1* (2.3 ± 0.2%), GCI/*SER3* (3.2 ± 0.2%)*,* GCI/*SER33* (2.9 ± 0.1%), GCI/*SER3*/*SER33* (2.5 ± 0.2%), and GCI/*SERs* (2.5 ± 0.2%) at 48 h were higher than that of the vector control GCI strain (1.6 ± 0.1%) (Fig. 3B). Because of the increased biomass concentration and intracellular glutathione content in the most of the recombinant strains, all constructed strains also showed improved volumetric glutathione production at 48 h compared to the GCI/Vector strain (17.9 ± 0.8 mg/L) (Fig. 3C). The volumetric glutathione production of the GCI/*SER2* (33.9 ± 3.9 mg/L), GCI/*SER1* (36.8 ± 1.3 mg/L), GCI/*SER3* (47.0 ± 3.2 mg/L)*,* GCI/*SER33* (47.7 ± 1.4 mg/L), and GCI/*SER3*/*SER33* (41.8 ± 4.5 mg/L) at 48 h were apparently higher than that of the vector control GCI strain (Fig. 3C). The GSSG ratio of GCI/*SER2* (26.1 ± 0.8%), GCI/*SER3* (32.0 ± 3.3%), and GCI/*SERs* (37.0 ± 5.6%) at 48 h were higher than that of the GCI/Vector strain (22.1 ± 0.6%). The GSSG ratio of GCI/*SER1* (20.7 ± 0.2%), GCI/*SER33* (23.6 ± 1.5%), and GCI/*SER3*/*SER33* (22.6 ± 0.9%) at 48 h were almost same to that of the GCI/Vector stain.

### Glutathione production by GCI/*SER3* and GCI/*SER33* strains engineered in Gly and/or l-Cys biosynthetic pathways

**Fig. 4:**
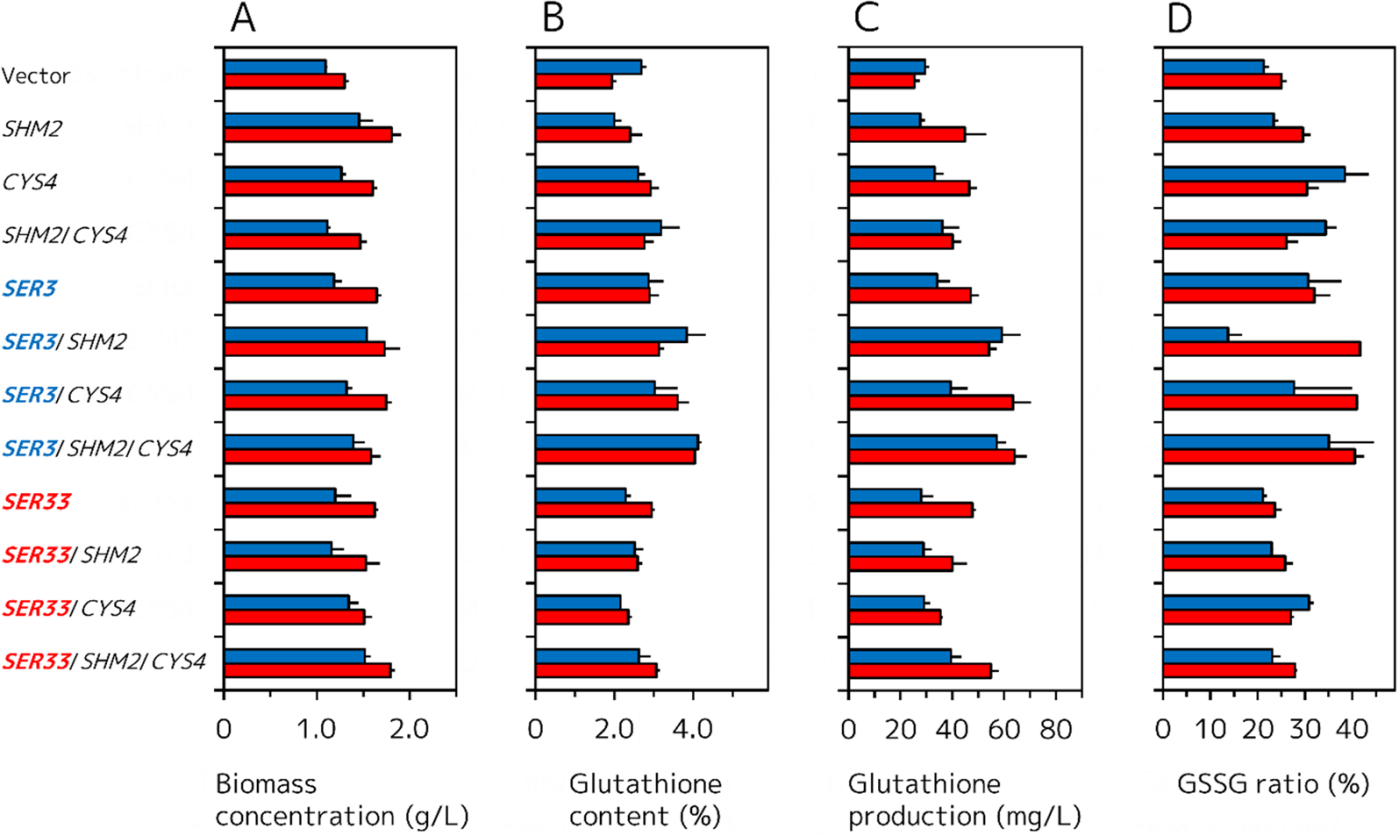
Glutathione production by GCI/Vector, GCI/*SER3*, and GCI/*SER33* strains overexpressing *SHM2* and/or *CYS4* genes. **A** Biomass concentration (g/L). **B** Intracellular glutathione content (%). **C** Volumetric glutathione production (mg/L). **D** GSSG ratio (%). *Blue* and *red filled bars* represent the values after growth for 24 and 48 h, respectively. Values are the mean, and error bars show the standard deviation (n = 3)

Enhancing l-Cys and Gly biosynthesis from l-Ser with enhancing l-Ser biosynthesis would be expected to further improve glutathione production by *S. cerevisiae*. l-Cys and a part of Gly are synthesized from l-Ser through reactions catalyzed by cystathionine γ-synthase (Cys4) and cytosolic serine hydroxymethyltransferase (Shm2), respectively (Fig. 1). In l-Ser biosynthetic genes, overexpression of *SER3* or *SER33* was effective to improve volumetric glutathione production (Fig. 3). Therefore, we constructed *SHM2* and/or *CYS4* overexpressing strains using GCI/Vector, GCI/*SER3*, and GCI/*SER33* as platform strains (Fig. 4). Unlike the case of the glutathione production by l-Ser biosynthetic gene overexpressing strains, the results of the strains overexpressing *SHM2* and/or *CYS4* using GCI/Vector, GCI/*SER3*, and GCI/*SER33* as platform strains were complicated. The biomass concentration, intracellular glutathione content, and volumetric glutathione production of GCI/*SHM2*, GCI/*CYS4*, and GCI/*SHM2*/*CYS4* at 48 h were apparently higher than those of the GCI/Vector (Fig. 4A–C; Additional file 1: Table S4). On the other hand, the GSSG ratio of the GCI/*SHM2* (29.6 ± 1.7%) and GCI/*CYS4* (30.4 ± 2.4%) at 48 h were somewhat higher than the that of the GCI/Vector (Fig. 4D), and the GSSG ratio of the GCI/*SHM2*/*CYS4* (26.1 ± 2.5%) at 48 h was almost same to the that of the GCI/Vector. These results indicate that overexpression of the *SHM2* and/or *CYS4* is effective for glutathione production by *S. cerevisiae* even when l-Ser biosynthetic pathway of the platform strain was not engineered. The intracellular glutathione content and volumetric glutathione production of the GCI/*SER3*/*SHM2*, GCI/*SER3*/*CYS4*, and GCI/*SER3*/*SHM2*/*CYS4* at 48 h were also higher than those of the platform strain GCI/*SER3* (Fig. 4B, C; Additional file 1: Table S4). However, the biomass concentration of the GCI/*SER3*/*SHM2* (1.73 ± 0.16 g/L) and GCI/*SER3*/*CYS4* (1.75 ± 0.06 g/L) at 48 h were slightly higher than that of the GCI/*SER3* (Fig. 4A), and that of the GCI/*SER3*/*SHM2*/*CYS4* (1.58 ± 0.11 g/L) was apparently decreased. Unlike using the GCI/Vector as a platform, the GSSG ratio of the GCI/*SER3*/*SHM2* (41.6 ± 0.2%), GCI/*SER3*/*CYS4* (41.0 ± 0.4%), and GCI/*SER3*/*SHM2*/*CYS4* (40.5 ± 2.0%) at 48 h were higher than that of the GCI/*SER3* (Fig. 4D). The biomass concentration, intracellular glutathione content, and volumetric glutathione production of the GCI/*SER33*/*SHM2* and GCI/*SER33*/*CYS4* at 48 h were obviously decreased compared to those of the platform strain GCI/*SER33*, whereas those of the GCI/*SER33*/*SHM2*/*CYS4* were higher than those of the GCI/*SER33* (Fig. 4A–C; Additional file 1: Table S4). Meanwhile, the GSSG ratio of the GCI/*SER33*/*SHM2* (25.8 ± 1.6%), GCI/*SER33*/*CYS4* (27.0 ± 0.7%), and GCI/*SER33*/*SHM2*/*CYS4* (27.9 ± 0.4%) at 48 h were slightly higher than that of the GCI/*SER3* (Fig. 4D).

### Glutathione production by GCI/*SER3*/*SER33* and GCI/*SERs* strains engineered in Gly and/or l-Cys biosynthetic pathways

**Fig. 5:**
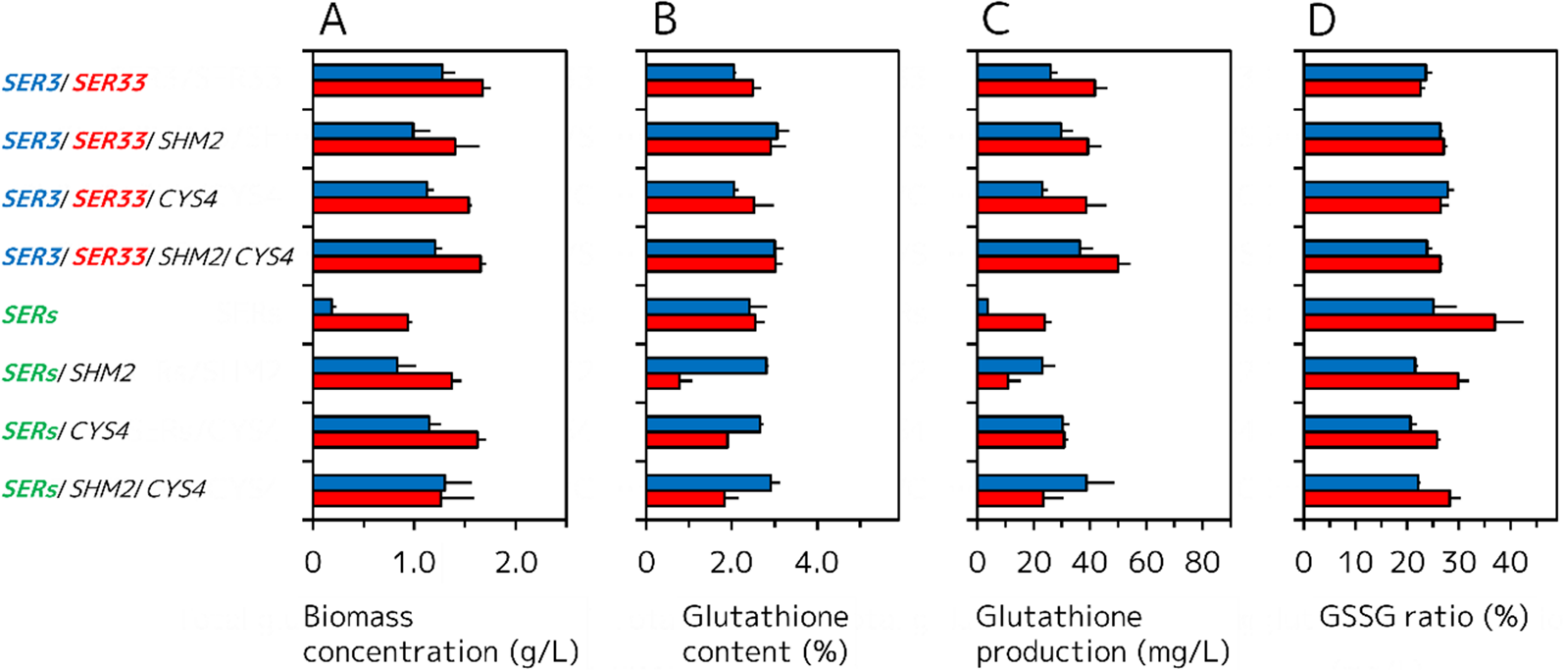
Glutathione production by GCI/*SER3*/*SER33* and GCI/*SERs* strains overexpressing *SHM2* and/or *CYS4* genes. **A** Biomass concentration (g/L). **B** Intracellular glutathione content (%). **C** Volumetric glutathione production (mg/L). **D** GSSG ratio (%). *Blue* and *red filled bars* represent the values after growth for 24 and 48 h, respectively. Values are the mean, and error bars show the standard deviation (n = 3)

As mentioned above, the intracellular glutathione content and volumetric glutathione production of the GCI/*SER3*/*SER33* and GCI/*SERs* were lower than those of the GCI/*SER3* and GCI/*SER33*. However, because we thought the cause of these low glutathione yields was excess l-Ser accumulation in cells by simultaneous overexpression of l-Ser biosynthetic genes, we constructed *SHM2* and/or *CYS4* overexpressing strains using the GCI/*SER3*/*SER33* and GCI/*SERs* as the platform strains and examined glutathione production of these strains (Fig. 5). The biomass concentration, intracellular glutathione content, and volumetric glutathione production of the GCI/*SER3*/*SER33*/*SHM2* and GCI/*SER3*/*SER33*/*CYS4* (Fig. 5A–C; Additional file 1: Table S5) were decreased or almost same than those of the platform GCI/*SER3*/*SER33* strain. On the other hand, although the GCI/*SER3*/*SER33*/*SHM2*/*CYS4* showed almost the same biomass concentration at 48 h (1.66 ± 0.06 g/L) (Fig. 5A) compared to that of the GCI/*SER3*/*SER33*, this strain showed higher intracellular glutathione content and volumetric glutathione production at 48 h (Fig. 5B, C; Additional file 1: Table S5). The GSSG ratio of the GCI/*SER3*/*SER33*/*SHM2*, GCI/*SER3*/*SER33*/*CYS4*, and GCI/*SER3*/*SER33*/*SHM2*/*CYS4* at 48 h (Fig. 5D; Additional file 1: Table S5) were higher than that of the GCI/*SER3*/*SER33* strain. The biomass concentration of the GCI/*SERs*/*SHM2* (1.37 ± 0.10 g/L), GCI/*SERs*/*CYS4* (1.54 ± 0.03 g/L), and GCI/*SERs*/*SHM2*/*CYS4* (1.66 ± 0.06 g/L) at 48 h were improved compared to that of the platform strain GCI/*SERs*. However, the intracellular glutathione content of the GCI/*SERs*/*SHM2* (0.8 ± 0.3%), GCI/*SERs*/*CYS4* (1.9 ± 0.1%), and GCI/*SERs*/*SHM2*/*CYS4* (1.8 ± 0.3%) at 48 h were lower than that of the GCI/*SERs*. Consequently, the volumetric glutathione production of the GCI/*SERs*/*SHM2* (10.9 ± 4.5 mg/L) and GCI/*SERs*/*SHM2*/*CYS4* (23.4 ± 7.2 mg/L) at 48 h were decreased, and the volumetric glutathione production of the GCI/*SERs*/*CYS4* (30.8 ± 1.4 mg/L) at 48 h was increased compared to that of the GCI/*SERs*. The GSSG ratio of the GCI/*SERs*/*SHM2* (29.9 ± 2.1%), GCI/*SERs*/*CYS4* (25.8 ± 0.6%), and GCI/*SERs*/*SHM2*/*CYS4* (28.3 ± 2.0%) at 48 h were lower than that of the GCI/*SERs*.

## Discussion

l-Ser biosynthesis from 3PG in *S. cerevisiae* occurs via three consecutive reactions catalyzed by *SER2*, *SER1*, and *SER3*/*SER33*. The intracellular glutathione content of the recombinant strains individually overexpressing *SER2, SER1, SER3*, and *SER33* were 1.4- to 1.8-fold higher than that of the control strain at 48 h (Fig. 3B). Albers et al. showed that transcription levels of *SER2*, *SER3*, and *SER33* were less than that of *SER1* [18]. The result that overexpression of *SER2*, *SER3*, and *SER33* increased intracellular glutathione content compared with that of *SER1* is likely primarily influenced by transcription levels of each gene. Furthermore, the result that the overexpression of *SER3* and *SER33* were the most effective to enhance the glutathione production (Fig. 3B, C) suggested that *SER3* and *SER33* were the most critical step in the l-Ser biosynthetic pathway, and this fact is consistent with the previous study which reported deletion of *SER3* and *SER33* equally influenced metabolite production [18]. Thus, we selected the GCI/*SER3* and GCI/*SER33* strains as the platform strain for further improvement of glutathione biosynthesis. However, simultaneous overexpression of *SER3* and *SER33* resulted in decreased intracellular glutathione content and volumetric glutathione production compared to those of the GCI/*SER3* and GCI/*SER33* strains (Fig. 3B, C). Furthermore, simultaneous overexpression of *SER2*, *SER1*, *SER3*, and *SER33* lead to poor growth and consecutive low glutathione production (Fig. 3A–C). These results may imply that surplus l-Ser production or decrease of 3PG by enhanced l-Ser biosynthesis exerts unfavorable effects on growth and glutathione production of host cells. The increase of GSSG ratio in the GCI/*SERs* strain also showed that the strain was under strong stress because GSSG ratio showed index of oxidative stress of cells [12–14]. However, the strains overexpressing *SER3* also showed high GSSG ratio compared with the control strain. In some cases, production of GSSG help to avoid a negative feedback regulation by Gsh1, and consequently increase total glutathione production [12–14]. The overexpression of *SER3* may lead modest stress of the cells for increased glutathione production. On the other hand, the strains overexpressing *SER33* showed slightly lower GSSG ratio than that of the control strains. These different effects of overexpression of *SER3* and *SER33* on GSSG ratios may show different mechanisms of improved glutathione production.

To further improve glutathione production by *S. cerevisiae*, we additionally overexpressed *SHM2* and/or *CYS4* genes encoding cytosolic serine hydroxymethyltransferase and cystathionine β-synthase, respectively (Fig. 1). Generally, cystathionine β-synthase encoded by *CYS4* catalyzes condensation of l-Ser and l-homocysteine to produce l-cystathionine and H_2_O. Consecutively, l-cystathionine is broken down into l-Cys, 2-oxobutanoic acid, and ammonia by cystathionine γ-lyase (*CYS3*). However, we didn’t adopt overexpression of *CYS3* in this study because Cys4 of *S. cerevisiae* can directly catalyze l-Ser and H_2_S to l-Cys and H_2_O [19] as shown in Fig. 1.

Unexpectedly, overexpression of *SHM2* and/or *CYS4* effectively enhanced biomass concentration, intracellular glutathione content, and consecutive volumetric glutathione production even when the GCI strain was used as a platform.

l-Cys is known as a late-limiting precursor for glutathione biosynthesis, and therefore a lot of studies have employed external addition of l-Cys to the culture medium for enhancing glutathione production [16, 17, 20–23]. Enhancing l-Cys biosynthesis with or without enhancing l-Ser biosynthesis also improved glutathione production of *S. cerevisiae* not only in this study, but also in previous study [11]. However, the result that enhancing Gly biosynthesis by genetic engineering on glutathione production has not been reported so far. According to the report of Wang et al., l-Glu abundantly exists in *S. cerevisiae* cells during fermentative glutathione production (185.9 ± 10 μmol/g-dry cell) [24] and intracellular Gly concentration (0.44 ± 0.15 μmol/g-dry cell) was almost the same as that of l-Cys (0.42 ± 0.2 μmol/g-dry cell) without addition of these precursors to the medium. In other cases, l-Cys concentration was lower than that of Gly; however, Gly concentration was much lower than that of l-Glu [25, 26]. Thus, there is a possibility that enhancing Gly biosynthesis promotes glutathione biosynthesis. In fact, addition of Gly to the medium improved glutathione production in a previous study [24]. On the other hand, Alfafara et al. reported that supplementation of Gly in the medium had no effect on the glutathione production of *S. cerevisiae* [16]. This disagreement may be caused by various factors of fermentation such as genotype of host *S. cerevisiae*, preculture conditions, initial cell concentration, medium composition, and so on. Indeed, even in this study, overexpression of *SHM2* in GCI/*SER33*, GCI/*SER3*/*SER33*, and GCI/*SERs* didn’t improve glutathione production (Fig. 4B, C).

Among the GCI/*SER3*/*SER33* and GCI/*SERs* strains overexpressing *SHM2* and/or *CYS4*, only GCI/*SER3*/*SER33*/*SHM2*/*CYS4* and GCI/*SERs*/*CYS4* showed modestly increased volumetric glutathione production compared to the platform strains (Fig. 5C). If the cause of poor glutathione production by GCI/*SER3*/*SER33* and GCI/*SERs* were surplus l-Ser biosynthesis, this disadvantage could be solved by overexpression of *SHM2* and/or *CYS4* regardless of the combination. However, the recovery of the cell growth by overexpression of *SHM2* and/or *CYS4* in GCI/*SERs* seemed to be the result that surplus biosynthesized l-Ser was metabolizing to Gly and l-Cys (Fig. 5A, D). However, overexpression of *SHM2* and/or *CYS4* had no effect or decreased cell growth when GCI/*SER3*/*SER33* was used as a platform (Fig. 5A). These results imply that there may be exquisite and complicated balance between supplies of l-Ser, l-Cys, and Gly, in enhanced glutathione production of *S. cerevisiae*. In this study, GCI/*SER3*/*SHM2*/*CYS4* showed the highest volumetric glutathione production at 48 h (64.0 ± 4.9 mg/L) among all strains constructed, and this yield is about 2.5-fold higher than that of the GCI/Vector strain. In other reports about fermentative glutathione production using yeasts, feeding of various precursors and carbon sources, and optimization of growth conditions have been often employed [23, 27–30]. In many cases adopting these strategies, the fermentation was carried out in nutrient rich condition with high cell density using glutathione high-producing strains and leached about 100–2500 mg/L of glutathione [31]. For example, Wen et al. reported 2190 mg/L of glutathione production in nutrient rich medium supplemented with l-Cys, l-Glu, and Gly using glutathione high-producing *S. cerevisiae* T65 strain under high cell density and glucose feeding conditions [28]. Comparing with glutathione production of these reports, the maximum glutathione production of this study (64.0 ± 4.9 mg/L) is very low. The *S. cerevisiae* strains in this study were grown in the nutrient limited minimal medium with low initial cell densities. These growth conditions may lead poor glutathione production compared with those of other reports. However, our objective of this study is to provide a new strategy that modification of l-Ser, l-Cys, and Gly biosynthetic genes improves glutathione production of *S. cerevisiae*. Indeed, glutathione production studies focused of genetic engineering reported about 10–300 mg/L of glutathione production [32] and the reason for these low yields may be the not optimized culture conditions. Furthermore, in many of these studies, genes directly involved in glutathione synthesis such as *GSH1* [33], *gshF* encoding bifunctional glutathione synthase [34], and *PRO1* encoding γ-glutamyl kinase [35] are overexpressed. In such situations, we focused on l-Ser biosynthetic genes and first revealed that engineering of l-Ser and Gly biosynthetic pathway are useful strategies for fermentative glutathione production by *S. cerevisiase*. These our findings may be applicable for other glutathione high-producing yeast strains to further improve glutathione production.

## Methods

### Strains and media

*Escherichia coli* NovaBlue strain (Novagen, Madison, WI, USA) was used as the host strain for recombinant DNA manipulation. *S. cerevisiae* GCI [*MATa ura3-52 lys2-801 ade2-101 trp1-Δ63 his3-Δ200 leu2-Δ1*], which was previously constructed from a YPH499 strain by expressing multiple copies of *GSH1* and *GSH2* genes by the δ-integration method [36], was used as the parental strain for additional overexpression of genes in this study. *E. coli* strains were grown in LB medium (10 g/L tryptone, 5 g/L yeast extract, and 5 g/L sodium chloride) supplemented with ampicillin (100 mg/L). Recombinant *S. cerevisiae* GCI strains were grown in SD (6.7 g/L yeast nitrogen base w/o amino acids, and 20 g/L glucose) medium. Adenine (20 mg/L), histidine (20 mg/L), tryptophan (40 mg/L), leucine (100 mg/L), uracil (20 mg/L) and aureobasidin A (0.5 mg/L) were supplemented as necessary.

### Plasmid construction and yeast transformation

Each gene involved in the l-Ser biosynthetic pathway (*SER2*, *SER1*, *SER3*, and *SER33*) were amplified by polymerase chain reaction (PCR) method from *S. cerevisiae* chromosomal deoxyribonucleic acid (DNA) using primer sets of SER2F1/R1, SER1F1/R1, SER3F1/R1, and SER33F1/R1, respectively (Additional file 1: Table S1). The amplified DNA fragments from the *SER2*, *SER1*, *SER3*, and *SER33* genes were inserted into the NotI site between *P*_*TDH3*_ and *T*_*TDH3*_ of pATP405 [37] to construct pATP405-*SER2*, pATP405-*SER1*, pATP405-*SER3*, and pATP405-*SER33*, respectively (Table 1). These constructed plasmids were then digested with EcoRV or AflII and transformed into *S. cerevisiae* GCI strain (Table 2).

**Table 1:** Plasmids used in this study

Plasmid	Description
pATP405	Intact vector
pATP405-*SER2*	*P*_*TDH3*_-*SER2*-*T*_*TDH3*_, *LEU2* marker
pATP405-*SER1*	*P*_*TDH3*_-*SER1*-*T*_*TDH3*_, *LEU2* marker
pATP405-*SER3*	*P*_*TDH3*_-*SER3*-*T*_*TDH3*_, *LEU2* marker
pATP405-*SER33*	*P*_*TDH3*_-*SER33*-*T*_*TDH3*_, *LEU2* marker
pATP405-*SER33*/*SER2*/*SER1*	*P*_*TDH3*_-*SER33*-*T*_*TDH3*_/*P*_*ADH1*_-*SER2*-*T*_*ADH1*_/*P*_*PGK1*_-*SER1*-*T*_*PGK1*_, *LEU2* marker
pATP406	Intact vector
pATP406-*SER3*	*P*_*PGK1*_-*SER3*-*T*_*PGK1*_, *URA3* marker
pATP406-*SHM2*	*P*_*PGK1*_-*SHM2*-*T*_*PGK1*_, *URA3* marker
pATP406-*CYS4*	*P*_*ADH1*_-*CYS4*-*T*_*ADH1*_, *URA3* marker
pATP406-*SHM2*/*CYS4*	*P*_*TDH3*_-*SHM2*-*T*_*TDH3*_/*P*_*ADH1*_-*CYS4*-*T*_*ADH1*_, *URA3* marker
pATP406-*SHM2*/*SER3*	*P*_*TDH3*_-*SHM2*-*T*_*TDH3*_/*P*_*PGK1*_-*SER3*-*T*_*PGK1*_, *URA3* marker
pATP406-*CYS4*/*SER3*	*P*_*TDH3*_-*CYS4*-*T*_*TDH3*_/*P*_*PGK1*_-*SER3*-*T*_*PGK1*_, *URA3* marker
pATP406-*SHM2*/*CYS4*/*SER3*	*P*_*TDH3*_-*SHM2*-*T*_*TDH3*_/*P*_*ADH1*_-*CYS4*-*T*_*ADH1*_/*P*_*PGK1*_-*SER3*-*T*_*PGK1*_, *URA3* marker

**Table 2:** Recombinant *S. cerevisiae* strains used in this study

Strain	Plasmid integrated in chromosome	Figure correspondence
*S. cerevisiae* GCI	Host strain	Figure 2
*S. cerevisiae* GCI/Vector	pATP405 and pATP406	Figures 3 and 4
*S. cerevisiae* GCI/*SER2*	pATP405-*SER2* and pATP406	Figure 3
*S. cerevisiae* GCI/*SER1*	pATP405-*SER1* and pATP406	Figure 3
*S. cerevisiae* GCI/*SER3*	pATP405-*SER3* and pATP406	Figures 3 and 4
*S. cerevisiae* GCI/*SER33*	pATP405-*SER33* and pATP406	Figures 3 and 4
*S. cerevisiae* GCI/*SER3*/*SER33*	pATP405-*SER33* and pATP406-*SER3*	Figures 3 and 5
*S. cerevisiae* GCI/*SERs*	pATP405-*SER33*/*SER2*/*SER1* and pATP406-*SER3*	Figures 3 and 5
*S. cerevisiae* GCI/*SHM2*	pATP405 and pATP406-*SHM2*	Figure 4
*S. cerevisiae* GCI/*CYS4*	pATP405 and pATP406-*CYS4*	Figure 4
*S. cerevisiae* GCI/*SHM2*/*CYS4*	pATP405 and pATP406-*SHM2*/*CYS4*	Figure 4
*S. cerevisiae* GCI/*SER3*/*SHM2*	pATP405-*SER3* and pATP406-*SHM2*	Figure 4
*S. cerevisiae* GCI/*SER3*/*CYS4*	pATP405-*SER3* and pATP406-*CYS4*	Figure 4
*S. cerevisiae* GCI/*SER3*/*SHM2*/*CYS4*	pATP405-*SER3* and pATP406-*SHM2*/*CYS4*	Figure 4
*S. cerevisiae* GCI/*SER33*/*SHM2*	pATP405-*SER33* and pATP406-*SHM2*	Figure 4
*S. cerevisiae* GCI/*SER33*/*CYS4*	pATP405-*SER33* and pATP406-*CYS4*	Figure 4
*S. cerevisiae* GCI/*SER33*/*SHM2*/*CYS4*	pATP405-*SER33* and pATP406-*SHM2*/*CYS4*	Figure 4
*S. cerevisiae* GCI/*SER3*/*SER33*/*SHM2*	pATP405-*SER33* and pATP406-*SHM2*/*SER3*	Figure 5
*S. cerevisiae* GCI/*SER3*/*SER33*/*CYS4*	pATP405-*SER33* and pATP406-*CYS4*/*SER3*	Figure 5
*S. cerevisiae* GCI/*SER3*/*SER33*/*SHM2*/*CYS4*	pATP405-*SER33* and pATP406-*SHM2*/*CYS4*/*SER3*	Figure 5
*S. cerevisiae* GCI/*SERs*/*SHM2*	pATP405-*SER33*/*SER2*/*SER1* and pATP406-*SHM2*/*SER3*	Figure 5
*S. cerevisiae* GCI/*SERs*/*CYS4*	pATP405-*SER33*/*SER2*/*SER1* and pATP406-*CYS4*/*SER3*	Figure 5
*S. cerevisiae* GCI/*SERs*/*SHM2*/*CYS4*	pATP405-*SER33*/*SER2*/*SER1* and pATP406-*SHM2*/*CYS4*/*SER3*	Figure 5

The genes involved in the Gly biosynthetic pathway (*SHM2*) and l-Cys biosynthetic pathway (*CYS4*) were amplified by PCR method from *S. cerevisiae* chromosomal DNA using primer sets of SHM2F/R, and CYS4F/R, respectively (Additional file 1: Table S1). The amplified DNA fragment from the *SHM2* gene was inserted into the NotI site of pATP406 [37] to construct pATP406-*SHM2* (Table 1). The amplified fragment from the *CYS4* gene was inserted into the PmeI site between *P*_*ADH1*_ and *T*_*ADH1*_ of pATP406 and pATP406-*SHM2* to construct pATP406-*CYS4* and pATP406-*SHM2*/*CYS4*, respectively (Table 1). These constructed plasmids were digested with *Nco*I, and transformed into appropriate *S. cerevisiae* host strains (Table 2).

To obtain recombinant strains simultaneously overexpressing all genes involved in the l-Ser, l-Cys and Gly biosynthetic pathways, *SER2*, *SER1,* and *SER3* genes were amplified by PCR method from *S. cerevisiae* chromosomal DNA using primer sets of SER2F2/R2, SER1F2/R2, and SER3F2/R2, respectively (Additional file 1: Table S1). The amplified DNA fragment from *SER2* was inserted into the PmeI site of pATP405-*SER33* to construct pATP405-*SER33*/*SER2* (Table 1). The amplified fragment from *SER1* was inserted into the AscI site between *P*_*PGK1*_ and *T*_*PGK1*_ of pATP405-*SER33*/*SER2* to construct pATP405-*SER33*/*SER2*/*SER1* (Table 1). The amplified fragment from *SER3* was inserted into the AscI site of pATP406, pATP406-*SHM2,* pATP406-*CYS4*, and pATP406-*SHM2*/*CYS4* to construct pATP406-*SER3*, pATP406-*SHM2*/*SER3*, pATP406-*CYS4*/*SER3*, and pATP406-*SHM2*/*CYS4*/*SER3*, respectively (Table 1). These plasmids were digested with EcoRI or NcoI, and transformed into appropriate *S. cerevisiae* host strains (Table 2).

Transformation of *S. cerevisiae* was carried out using the lithium acetate method as described previously [38, 39]. Transformants were selected by leucine (pATP405) and uracil (pATP406) auxotrophies. The insertion of the target gene in each transformant was confirmed by PCR method using appropriate primers.

### Glutathione production using* S. cerevisiae* mutant strains

Glycerol stocks of recombinant *S. cerevisiae* strains stored at −80 °C were streaked on solid SD medium and aerobically grown for 72 h. Grown cells on solid media were inoculated into 5 mL of the liquid SD medium and aerobically grown at 30 °C with agitation (200 rpm) for 24 h. An adequate volume of each cell culture was inoculated into 20 mL of the same medium in 200 mL of baffled erlenmeyer flask with or without 300 mg/L l-Ser to achieve an initial OD_600_ value of 0.15. Cells were then grown at 30 °C with agitation (200 rpm) for up to 72 h.

### Glutathione analysis

Reduced glutathione (GSH) and oxidized glutathione (GSSG) in recombinant *S. cerevisiae* cells were determined as described in our previous report [40]. Cell cultures (1 mL) grown for glutathione production were sampled every 24 h, and the OD_600_ of the culture samples were measured using a UVmini-1240 Spectrometer (Shimadzu, Kyoto, Japan). The cells were pelleted by centrifugation (16,000×*g*, 1 min) and rinsed with Milli-Q water twice. The rinsed cell pellets were re-suspended in Milli-Q water and incubated at 95 °C for 3 min, then cooled immediately on ice for 3 min and the supernatant was separated by centrifugation (16,000×*g*, 1 min). GSH and GSSG concentrations in the supernatant were determined by HPLC (Shimadzu) equipped with a YMC-Pack ODS-A column (YMC, Kyoto, Japan). The operating conditions were 30 °C with 50 mM potassium dihydrogen phosphate buffer (pH 2.8) and 10 mM sodium 1-heptanesulfonate as the mobile phase at a flow rate of 1.0 mL/min, and detection was performed with an ultraviolet detector SPD-20A (Shimadzu) at 210 nm. Volumetric glutathione production (mg/L-broth) and intracellular glutathione content (%) were calculated from OD_600_ and used to determine glutathione concentration, as previously described [40].

## Supplementary Information

**Additional file 1: Table S1.** Primers used in this study. **Table S2**. Data used in Fig. 2.** Table S3** Data used in Fig. 3.** Table S4** Data used in Fig. 4.** Table S5** Data used in Fig. 5.

## Data Availability

All data of figures used in this study are included in Additional files of this article.

## Abbreviations

2OG: 2-Oxoglutarate
3PG: 3-Phospho-glycerate
3PHP: 3-Phospho-hydroxypyruvate
5,10-CH_2_-THF: (6R)-5,10-methylenetetrahydrofolate
ADP: Adenosine diphosphate
ATP: Adenosine triphosphate
DNA: Deoxyribonucleic acid
γ-GC: γ-Glutamylcysteine
GCS: Glutamylcysteine synthetase encoded
Gly: Glycine
GS: Glutathione synthetase
GSH: Reduced glutathione
GSSG: Oxidized glutathione
H_2_S: Hydrogen sulfide
l-Cys: L-cysteine
l-Glu: L-glutamine
LPS: L-*O*-phosphoserine
l-Ser: L-serine
NAD^+^: Nicotinamide adenine dinucleotide
NADH: Reduced form of nicotinamide adenine dinucleotide
PCR: Polymerase chain reaction
Pi: Phosphate
THF: Tetrahydrofolate
