# Supplementary material for: Metabolic engineering of the l-serine biosynthetic pathway improves glutathione production in Saccharomyces cerevisiae
Source: Microb Cell Fact. 2022 Aug 6;21:153. doi: 10.1186/s12934-022-01880-8 (PMC9356478; doi:10.1186/s12934-022-01880-8)
Supplement: Supplementary file 1 — Additional file 1: Table S1. Primers used in this study. Table S2. Data used in Fig. 2. Table S3 Data used in Fig. 3. Table S4 Data used in Fig. 4. Table S5 Data used in Fig. 5. [file 12934_2022_1880_MOESM1_ESM.docx]

Submitted to *Microbial Cell Factories*

**Title:** Overexpression of L-serine, L-cysteine, and glycine biosynthesis genes increases glutathione production in *Saccharomyces cerevisiae*

**Authors:** Jyumpei Kobayashi^1^, Daisuke Sasaki^1^, Kiyotaka Y. Hara^1, 2^, Tomohisa Haunuma^1,3^, Akihiko Kondo^1, 3,4^

**Author detailed**

^1^Graduate School of Science, Technology and Innovation, Kobe University, 1-1 Rokkodaicho, Nada-ku, Kobe, Hyogo 657-8501, Japan

^2^Graduate School of Nutritional and Environmental Sciences, University of Shizuoka, 52‑1 Yada, Suruga‑ku, Shizuoka 422‑8526, Japan

^3^Engineering Biology Research Center, Kobe University, 1-1 Rokkodaicho, Nada-ku, Kobe, Hyogo 657-8501, Japan

^4^RIKEN Center for Sustainable Resource Science, 1-7-22 Suehiro-cho, Tsurumi-ku, Yokohama, Kanagawa 230-0045, Japan

**Corresponding author:**

Mailing address: Department of Chemical Science and Engineering, Graduate School of Engineering, Kobe University, 1-1 Rokkodaicho, Nada-ku, Kobe 657-8501, Japan. Phone and fax: 81-78-803-6196. E-mail: akondo@kobe-u.ac.jp

| Primer | Sequence (5'-3') | Cloning site |
| --- | --- | --- |
| SER2F1 | GACACGCGTGCGGCCATGTCAAAGTTTGTTATCAC | NotI site between *P_TDH3_* and *T_TDH3_* |
| SER2R1 | TAAATTCACGCGGCCTCATTGTCTATTGTATATTTC | NotI site between *P_TDH3_* and *T_TDH3_* |
| SER2F2 | CGCGGCCGGCCGTTTTCATTGTCTATTGTATATTTC | PmeI site between *P_ADH1_* and *T_ADH1_* |
| SER2R2 | ATACACCTAGGGTTTATGTCAAAGTTTGTTATCAC | PmeI site between *P_ADH1_* and *T_ADH1_* |
| SER1F1 | GTCGACACGCGTGCGGCCATGTCTTTGGAAAGAGAGGAAC | NotI site between *P_TDH3_* and *T_TDH3_* |
| SER1R1 | TAAATTCACGCGGCCTTAAGCGTTTTTCTCAGCAAATTC | NotI site between *P_TDH3_* and *T_TDH3_* |
| SER1F2 | AACCCCGGGGGCGCGATGTCTTTGGAAAGAGAGGAAC | AscI site between *P_PGK1_* and *T_PGK1_* |
| SER1R2 | ATTTATTTCGGCGCGTTAAGCGTTTTTCTCAGC | AscI site between *P_PGK1_* and *T_PGK1_* |
| SER3F1 | GACACGCGTGCGGCCATGACAAGCATTGACATTAAC | NotI site between *P_TDH3_* and *T_TDH3_* |
| SER3R1 | GTAAATTCACGCGGCCTTAATATAGCAATCTAATTG | NotI site between *P_TDH3_* and *T_TDH3_* |
| SER3F2 | AACCCCGGGGGCGCGATGACAAGCATTGACATTAAC | AscI site between *P_PGK1_* and *T_PGK1_* |
| SER3R2 | ATTTATTTCGGCGCGTTAATATAGCAATCTAATTG | AscI site between *P_PGK1_* and *T_PGK1_* |
| SER33F | GACACGCGTGCGGCCATGTCTTATTCAGCTGCCG | NotI site between *P_TDH3_* and *T_TDH3_* |
| SER33R | TAAATTCACGCGGCCTTAGTATAATAACCTGATGG | NotI site between *P_TDH3_* and *T_TDH3_* |
| SHM2F | GACACGCGTGCGGCCATGCCTTACACTCTATCCGAC | NotI site between *P_TDH3_* and *T_TDH3_* |
| SHM2R | TAAATTCACGCGGCCTTACACAGCCAATGGGTATTC | NotI site between *P_TDH3_* and *T_TDH3_* |
| CYS4F | ATACACCTAGGGTTTATGCTAAGTAGCTCAGTAAATC | PmeI site between *P_ADH1_* and *T_ADH1_* |
| CYS4R | ATACACCTAGGGTTTTATGACTAAATCTGAGCAGCAAG | PmeI site between *P_ADH1_* and *T_ADH1_* |

Table S1 Primers used in this study

|  | Biomass concentration (g/L) | |  | Intracellular glutathione content (%) | |  | Volumetric glutathione production (mg/L) | |
| --- | --- | --- | --- | --- | --- | --- | --- | --- |
| Incubation time (h) | SD medium | SD medium supplemented with L-Ser |  | SD medium | SD medium supplemented with L-Ser |  | SD medium | SD medium supplemented with L-Ser |
| 0 | 0.04 | 0.04 |  | 2.4 | 2.4 |  | 0.9 | 0.9 |
| 24 | 1.27 ± 0.03 | 1.38 ± 0.06 |  | 2.7 ± 0.1 | 2.6 ± 0.1 |  | 34.6 ± 0.9 | 35.3 ± 1.2 |
| 48 | 1.37 ± 0.05 | 1.45 ± 0.03 |  | 2.2 ± 0.1 | 2.5 ± 0.2 |  | 30.7 ± 2.5 | 36.6 ± 1.2 |
| 72 | 1.42 ± 0.03 | 1.65 ± 0.05 |  | 1.5 ± 0.1 | 2.2 ± 0.1 |  | 20.7 ± 1.9 | 36.6 ± 0.7 |

Table S2 Data used in Fig. 2

Table S3 Data used in Fig. 3

|  | Incubation time (h) | Biomass concentation (g/L) | Volumetic glutathione production (mg/L) | Intracellular glutathione content (%) | GSSG ratio (%) |
| --- | --- | --- | --- | --- | --- |
| GCI/Vector | 24 | 1.09 ± 0.02 | 29.4 ± 1.5 | 2.7 ± 0.1 | 21.2 ± 1.2 |
| GCI/*SER2* | 24 | 1.10 ± 0.02 | 26.6 ± 1.3 | 2.4 ± 0.1 | 18.0 ± 0.6 |
| GCI/*SER1* | 24 | 1.23 ± 0.08 | 20.1 ± 0.6 | 1.6 ± 0.1 | 18.4 ± 0.5 |
| GCI/*SER3* | 24 | 1.18 ± 0.09 | 34.1 ± 5.1 | 2.9 ± 0.4 | 30.7 ± 7.0 |
| GCI/*SER33* | 24 | 1.20 ± 0.17 | 28.0 ± 4.6 | 2.3 ± 0.1 | 21.1 ± 0.9 |
| GCI/*SER3*/*SER33* | 24 | 1.28 ± 0.12 | 26.0 ± 2.6 | 2.0 ± 0.1 | 23.6 ± 1.3 |
| GCI/*SERs* | 24 | 0.18 ± 0.04 | 3.7 ± 0.5 | 2.4 ± 0.4 | 25.1 ± 4.5 |
| GCI/Vector | 48 | 1.30 ± 0.05 | 25.4 ± 2.2 | 1.9 ± 0.1 | 25.0 ± 1.1 |
| GCI/*SER2* | 48 | 1.22 ± 0.03 | 33.9 ± 1.3 | 2.8 ± 0.1 | 26.1 ± 0.2 |
| GCI/*SER1* | 48 | 1.57 ± 0.01 | 36.8 ± 3.9 | 2.3 ± 0.3 | 20.7 ± 0.8 |
| GCI/*SER3* | 48 | 1.64 ± 0.05 | 47.0 ± 3.2 | 2.9 ± 0.2 | 32.0 ± 3.3 |
| GCI/*SER33* | 48 | 1.62 ± 0.04 | 47.7 ± 1.4 | 2.9 ± 0.1 | 23.6 ± 1.5 |
| GCI/*SER3*/*SER33* | 48 | 1.67 ± 0.08 | 41.8 ± 4.5 | 2.5 ± 0.2 | 22.6 ± 0.9 |
| GCI/*SERs* | 48 | 0.94 ± 0.04 | 24.0 ± 2.4 | 2.5 ± 0.2 | 37.0 ± 5.6 |

Table S4 Data used in Fig. 4

|  | Incubation time (h) | Biomass concentation (g/L) | Volumetic glutathione production (mg/L) | Intracellular glutathione content (%) | GSSG ratio (%) |
| --- | --- | --- | --- | --- | --- |
| GCI/Vector | 24 | 1.09 ± 0.02 | 29.4 ± 1.5 | 2.7 ± 0.1 | 21.2 ± 1.2 |
| GCI/*SHM2* | 24 | 1.45 ± 0.16 | 27.6 ± 1.9 | 2.0 ± 0.2 | 23.4 ± 1.1 |
| GCI/*CYS4* | 24 | 1.27 ± 0.05 | 33.2 ± 3.3 | 2.6 ± 0.2 | 38.4 ± 5.1 |
| GCI/*SHM2*/*CYS4* | 24 | 1.11 ± 0.04 | 36.2 ± 6.4 | 3.2 ± 0.5 | 34.4 ± 2.3 |
| GCI/*SER3* | 24 | 1.18 ± 0.09 | 34.1 ± 5.1 | 2.9 ± 0.4 | 30.7 ± 7.0 |
| GCI/*SER3*/*SHM2* | 24 | 1.54 ± 0.07 | 59.0 ± 7.2 | 3.8 ± 0.5 | 13.7 ± 2.9 |
| GCI/*SER3*/*CYS4* | 24 | 1.32 ± 0.07 | 39.4 ± 6.4 | 3.0 ± 0.6 | 27.7 ± 12.2 |
| GCI/*SER3*/*SHM2*/*CYS4* | 24 | 1.39 ± 0.12 | 57.1 ± 3.6 | 4.1 ± 0.1 | 35.1 ± 9.6 |
| GCI/*SER33* | 24 | 1.20 ± 0.17 | 28.0 ± 4.6 | 2.3 ± 0.1 | 21.1 ± 0.9 |
| GCI/*SER33*/*SHM2* | 24 | 1.16 ± 0.13 | 28.8 ± 3.3 | 2.5 ± 0.2 | 23.0 ± 0.4 |
| GCI/*SER33*/*CYS4* | 24 | 1.34 ± 0.11 | 29.1 ± 2.5 | 2.2 ± 0.1 | 30.8 ± 1.1 |
| GCI/*SER33*/*SHM2*/*CYS4* | 24 | 1.52 ± 0.06 | 39.6 ± 4.1 | 2.6 ± 0.3 | 23.1 ± 1.8 |
| GCI/Vector | 48 | 1.30 ± 0.05 | 25.4 ± 2.2 | 1.9 ± 0.1 | 25.0 ± 1.1 |
| GCI/*SHM2* | 48 | 1.81 ± 0.10 | 44.8 ± 8.4 | 2.4 ± 0.3 | 29.6 ± 1.7 |
| GCI/*CYS4* | 48 | 1.60 ± 0.04 | 46.6 ± 3.0 | 2.9 ± 0.2 | 30.4 ± 2.4 |
| GCI/*SHM2*/*CYS4* | 48 | 1.47 ± 0.07 | 40.2 ± 3.2 | 2.8 ± 0.2 | 26.1 ± 2.5 |
| GCI/*SER3* | 48 | 1.64 ± 0.05 | 47.0 ± 3.2 | 2.9 ± 0.2 | 32.0 ± 3.3 |
| GCI/*SER3*/*SHM2* | 48 | 1.73 ± 0.16 | 54.1 ± 3.1 | 3.1 ± 0.1 | 41.6 ± 0.2 |
| GCI/*SER3*/*CYS4* | 48 | 1.75 ± 0.06 | 63.4 ± 7.1 | 3.6 ± 0.3 | 41.0 ± 0.4 |
| GCI/*SER3*/*SHM2*/*CYS4* | 48 | 1.58 ± 0.11 | 64.0 ± 4.9 | 4.0 ± 0.1 | 40.5 ± 2.0 |
| GCI/*SER33* | 48 | 1.62 ± 0.04 | 47.7 ± 1.4 | 2.9 ± 0.1 | 23.6 ± 1.5 |
| GCI/*SER33*/*SHM2* | 48 | 1.53 ± 0.15 | 40.0 ± 5.7 | 2.6 ± 0.1 | 25.8 ± 1.6 |
| GCI/*SER33*/*CYS4* | 48 | 1.51 ± 0.08 | 35.4 ± 1.1 | 2.4 ± 0.1 | 27.0 ± 0.7 |
| GCI/*SER33*/*SHM2*/*CYS4* | 48 | 1.79 ± 0.05 | 54.9 ± 3.0 | 3.1 ± 0.1 | 27.9 ± 0.4 |

Table S5 Data used in Fig. 5

|  | Incubation time (h) | Biomass concentation (g/L) | Volumetic glutathione production (mg/L) | Intracellular glutathione content (%) | GSSG ratio (%) |
| --- | --- | --- | --- | --- | --- |
| GCI/*SER3*/*SER33* | 24 | 1.28 ± 0.12 | 26.0 ± 2.6 | 2.0 ± 0.1 | 23.6 ± 1.3 |
| GCI/*SER3*/*SER33*/*SHM2* | 24 | 0.99 ± 0.17 | 29.7 ± 4.3 | 3.1 ± 0.3 | 26.4 ± 0.6 |
| GCI/*SER3*/*SER33*/*CYS4* | 24 | 1.13 ± 0.07 | 23.0 ± 2.0 | 2.0 ± 0.1 | 27.9 ± 1.2 |
| GCI/*SER3*/*SER33*/*SHM2*/*CYS4* | 24 | 1.21 ± 0.07 | 36.5 ± 4.6 | 3.0 ± 0.2 | 23.8 ± 1.1 |
| GCI/*SERs* | 24 | 0.18 ± 0.04 | 3.7 ± 0.5 | 2.4 ± 0.4 | 25.1 ± 4.5 |
| GCI/*SERs*/*SHM2* | 24 | 0.83 ± 0.19 | 23.1 ± 4.8 | 2.8 ± 0.1 | 21.5 ± 0.5 |
| GCI/*SERs*/*CYS4* | 24 | 1.15 ± 0.12 | 30.3 ± 2.3 | 2.7 ± 0.1 | 20.7 ± 1.2 |
| GCI/*SERs*/*SHM2*/*CYS4* | 24 | 1.30 ± 0.27 | 38.8 ± 9.8 | 2.9 ± 0.2 | 22.2 ± 0.4 |
| GCI/*SER3*/*SER33* | 48 | 1.67 ± 0.08 | 41.8 ± 4.5 | 2.5 ± 0.2 | 22.6 ± 0.9 |
| GCI/*SER3*/*SER33*/*SHM2* | 48 | 1.40 ± 0.24 | 39.3 ± 5.0 | 2.9 ± 0.4 | 27.1 ± 0.7 |
| GCI/*SER3*/*SER33*/*CYS4* | 48 | 1.54 ± 0.03 | 38.7 ± 7.3 | 2.5 ± 0.5 | 26.5 ± 1.7 |
| GCI/*SER3*/*SER33*/*SHM2*/*CYS4* | 48 | 1.66 ± 0.06 | 50.1 ± 4.3 | 3.0 ± 0.2 | 26.4 ± 0.6 |
| GCI/*SERs* | 48 | 0.94 ± 0.04 | 24.0 ± 2.4 | 2.5 ± 0.2 | 37.0 ± 5.6 |
| GCI/*SERs*/*SHM2* | 48 | 1.37 ± 0.10 | 10.9 ± 4.5 | 0.8 ± 0.3 | 29.9 ± 2.1 |
| GCI/*SERs*/*CYS4* | 48 | 1.62 ± 0.08 | 30.8 ± 1.4 | 1.9 ± 0.1 | 25.8 ± 0.6 |
| GCI/*SERs*/*SHM2*/*CYS4* | 48 | 1.26 ± 0.33 | 23.4 ± 7.2 | 1.8 ± 0.3 | 28.3 ± 2.0 |
